# Antipruritic effect of cold stimulation at the Quchi Acupoint (LI11) in Mice

**DOI:** 10.1186/1472-6882-14-341

**Published:** 2014-09-19

**Authors:** Kao-Sung Tsai, Yung-Hsiang Chen, Huey-Yi Chen, Ein-Yiao Shen, Yu-Chen Lee, Jui-Lung Shen, San-Yuan Wu, Jaung-Geng Lin, Yi-Hung Chen, Wen-Chi Chen

**Affiliations:** School of Chinese Medicine, Graduate Institute of Integrated Medicine, Graduate Institute of Acupuncture Science, College of Chinese Medicine, Research Center for Chinese Medicine & Acupuncture, China Medical University, Taichung, Taiwan; Division of Chinese Acupuncture, Departments of Dermatology, Medical Research, Obstetrics and Gynecology, and Urology, China Medical University Hospital, Taichung, Taiwan; Department of Applied Cosmetology, Master Program of Cosmetic Science, Hungkuang University, Taichung, Taiwan; Department of Dermatology, Taichung Veteran General Hospital, Taichung, Taiwan; Center for General Education, Feng Chia University, Taichung, Taiwan

**Keywords:** Acupuncture, Cold stimulation, Itch, Quchi (LI11), Transient receptor potential

## Abstract

**Background:**

Acupuncture and moxibustion are used to treat pruritus and atopic dermatitis. However, whether cold stimulation (defined as that the temperature conducted under skin temperature) of acupoints affects itching in experimental murine models remains unclear.

**Methods:**

The present study was designed to determine the therapeutic effects of different thermal stimulations at the Quchi acupoint (LI11) in a murine model in which scratching behaviour was elicited by subcutaneous injection with a pruritogenic agent (compound 48/80). Male ICR mice were divided into several groups as follows: control (saline), those receiving compound 48/80 and compound 48/80 with various thermal stimulations (5°C–45°C) at LI11 (n = 6 per group). The scratch response of each animal to these stimulations was recorded for 30 min. The antipruritic effect of the acupoint was further evaluated in LI11 and sham (non-acupoint) groups (n = 6 per group).

**Results:**

Treatment with lower temperature (20°C) at the LI11 acupoint significantly attenuated compound 48/80-induced scratching; however, this antipruritic effect was not observed with stimulation at the sham point. The expression of c-fos in the neuron of the cervical spine induced by compound 48/80 was suppressed by cold stimulation at LI11. The antipruritic effect of cold stimulation was blocked by ruthium red (RR), a non-selective transient receptor potential (TRP) channel blocker, suggesting that TRP channels may play an important role in the antipruritic effect of cold stimulation at LI11 in mice.

**Conclusions:**

This study demonstrated that cold stimulation at LI11 attenuated compound 48/80-induced scratching behaviour in mice, possibly by a TRP-related pathway.

## Background

Acupoint stimulation has been practiced in traditional Chinese medicine for over 2,500 years [1–3]. Several clinical trials have showed that physical stimulation, such as that with needles [4], moxibustion [a traditional Chinese medicine therapy burning moxa made from dried mugwort (*Artemisia argyi*)], or pressing, at the correct acupoints significantly decrease pruritus [5–8]. Much clinical and pre-clinical data attest to the fact that acupoint stimulation results in multiple biological responses [9–11]. Several clinic trials of histamine-dependent pruritus [12], allergic skin itch [13–15], neurogenic pruritus [16] and refractory uremic pruritus [17] in patients have shown that pruritus is significantly decreased by acupuncture treatment [18–20]. Accordingly, previous studies have shown that Quchi (LI11) is the acupoint commonly used for the treatment of pruritus [5, 17].

Compound 48/80 is well known as a peripheral pruritogen. It produces an itch sensation and vigorous scratching behaviour after subcutaneous (s.c.) injection; the itching then causes the degranulation of mast cells and the stimulation of leukotriene B4 production [21]. However, compound 48/80 also elicited the itch sensation in mast cell-deficient mice. Thus, it appears that compound 48/80 induces scratching behaviour by a mast cell-independent pathway [22]. Moreover, compound 48/80-induced scratching behaviour was inhibited by intrathecal injection with a gastrin releasing peptide receptor antagonist [23]. Compound 48/80 was applied to the murine model used in this study because this experimental system has been verified and is now established as a standard method for evaluating the effect of antiallergic treatment [24–26].

Although acupuncture or moxibustion has been practiced for over 2,500 years, the cooling device was invented only in recent 200 years and was unavailable for the traditional medical treatment. Thus, there were no relevant reports and researches of cold stimulation of the acupoints was addressed. The aim of the present study was to characterise the antipruritic effect of different thermal stimulations at LI11 on compound 48/80-induced scratching behaviour in the murine model. The experiment to investigate this was performed using different thermal stimulations. To explore any other viable treatments, in this study, we also assessed whether pre-treatment at LI11 with ruthenium red [RR; a non-selective transient receptor potential (TRP) channel blocker] would alter antipruritic efficacy in mice. Furthermore, the effect of thermal stimulation with 20°C at LI11 on neuronal activity and c-fos expression in the cervical spinal cord was investigated using immunohistochemistry (IHC).

## Methods

### Animals

**Figure 1:**
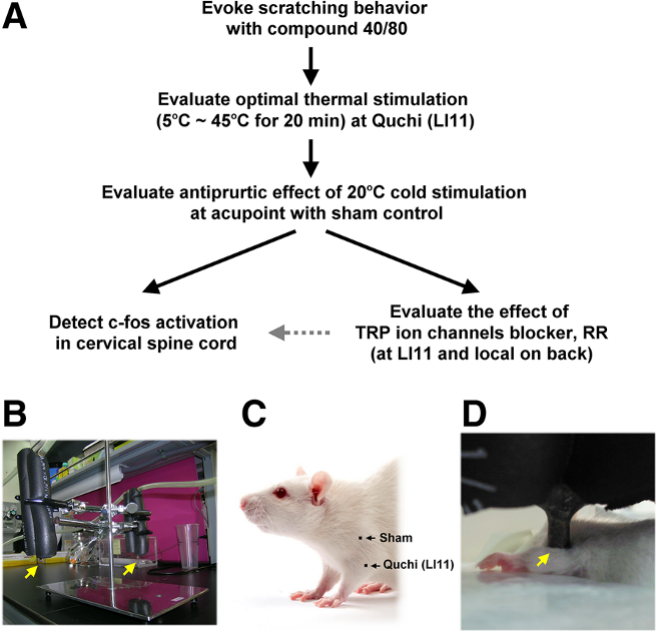
**Evaluating the antipruritic effect of different thermal stimulations over Quchi (LI11) acupoint on compound 48/80-induced scratching behavior. (A)** Flow chart depicting steps involved in the study. **(B)** The probes (arrows) of constant temperature tissue cooling apparatus. **(C)** Localization and **(D)** operation of LI11 acupoint stimulated in the mice. It is located at the depression medial to the extensor carpi radialis, at the lateral end of the cubital crease. The sham point was located at the midpoint of the acromial part of the deltoid muscle, which is distant from classical acupoints.

ICR mice (25–30 g, BioLasco Taiwan Co., Ltd., Taiwan) were applied to this study. Mice were maintained at a stable room temperature (22°C ± 1°C), humidity (50%–75%) and intermittent 12-h light and dark cycles for 20 h. Food and water were available ad libitum in the animal facility for at least 4 days before the experiments. The experimental procedures (Figure 1A) were approved by the China Medical University Institutional Animal Care and Use Committee in accordance with the care and use of laboratory animal guidebook from the Chinese Taipei Society of Laboratory Animal Sciences.

### Effect of thermal stimulation at the LI11 acupoint on compound 48/80-induced scratching behaviour

Fixed s.c. doses of compound 48/80 (10 mg/kg) were used to induce scratching. Compound 48/80 was purchased from Sigma Chemical Co. (St. Louis, MO, USA). Male ICR mice were randomised into several groups (n = 6 per group) as follows: control (saline) without thermal stimulation, those receiving compound 48/80 (s.c.) without thermal stimulation and those receiving compound 48/80 with different thermal stimulations (5°C–45°C) at LI11. The “cold stimulation” is defined as that the temperature conducted under skin temperature, in the other hand the “warm stimulation” is the temperature conducted over skin temperature [27]. The average skin temperature over LI11 of mice in this present study was 28.9°C ± 1.1°C.

Mice were individually acclimated in rectangular observation boxes for at least 1 h and then anesthetised with isoflurane. Different thermal stimulations were performed at LI11 using a constant temperature tissue cooling apparatus (Z3008, Taiwan Advanced Sterilization Technologies Inc., Taiwan) (Figure 1B) for 20 min under anaesthesia. There was only mild erythema in the site on mouse skin after 5°C or 45°C thermal stimulation. This mild erythema was recovered after one day. Murine LI11 is located at the depression medial to the extensor carpi radialis brevis (at the lateral end of the cubital crease), the equivalent of the human Quchi acupoint (Figures 1C and 1D). After different thermal stimulations, mice were allowed to recover from anaesthesia for 30 min and were then injected subcutaneously with compound 48/80 on the midline of the neck. The mice were videotaped after injected compound 48/80 over upper back for last 30 minutes. Videotapes were reviewed by investigators blinded to treatment. Animals were videotaped and the number of hind leg scratches directed to the back of the neck was counted by another investigator blinded to treatment. Bouts of scratching directed toward the injection site were counted in 5 min intervals for total 30 min. Hind paw movements directed away from the injection site (e.g. ear-scratching), licking or biting of the toes and grooming movements were not counted. We used both parameters, within-bout scratching frequency and number of scratch bouts, however, only total counts of scratch bouts were significantly correlated with the concentration of the pruritic stimulus [28].

### Cold stimulation at LI11 and a sham point (non-acupoint)

Twenty-four mice were further divided into four groups (n = 6 per group) as follows: control, those receiving compound 40/80 without thermal stimulation and with cold (20°C) stimulation at LI11 and cold stimulation at sham (non-acupoint). After thermal stimulation and recovery from anesthesia for 30 min, these mice were injected with compound 48/80 on the midline of the neck. The number of scratches to the back of the neck was counted for 30 min. The sham point was located at the midpoint of the acromial part of the deltoid muscle, which is distant from classical acupoints (Figure 1C) [29].

### Alteration of neural activity in the cervical spinal cord after 20°C thermal stimulation at LI11

Compound 48/80 induced c-fos expression in the dorsal horn of the cervical spinal cord. IHC was performed as described previously [30, 31]. Two hours after injection with compound 48/80, animals were anesthetised using urethane [1.2 g/kg intraperitoneal (i.p.)]. The cervical spinal cord was then removed and fixed in 4% paraformaldehyde solution overnight at 4°C. Tissue samples were transferred to 30% sucrose solution for at least 3–4 days before sectioning.

Slides were stained with c-fos (Bioss, Woburn, MA, USA) polyclonal antibodies using a Bond-Max autostainer (Leica Microsystems, Singapore). In brief, formalin-fixed and paraffin-embedded tissue array specimens were washed in Tris-buffered saline and Tween 20, rehydrated with serial dilutions of alcohol and washed in phosphate-buffered saline (PBS; pH 7.2, which was also used for all subsequent washes) according to the manufacturer’s recommended protocol. Slides were stained with previously mentioned antibodies using the fully automated Bond-Max system with heat-induced antigen retrieval and a VBS Refine polymer detection system (Leica Microsystems, Singapore). Diaminobenzidine was used as the chromogen for all IHC reactions [32, 33]. Negative controls were obtained by excluding the primary antibody [34, 35]. The c-fos-positive neurons were observed under a light microscope at high-power field (HPF) magnification [36], and the average number of c-fos-positive neurons was counted single-blind using the imaging software (Image-Pro Plus, Media Cybernetics, Inc., USA).

### Effect of the TRP ion channel blocker on thermal stimulation at LI11

The non-selective TRP ion channel blocker RR was injected s.c. (0.5 μmol/site) at LI11 5 min before 20°C stimulation. After thermal stimulation and recovery from anaesthesia for 30 min, these mice were injected with compound 48/80 on the midline of the neck. The number of scratches to the back of the neck was counted for 30 min.

### Data analysis

Statistical comparisons were performed using one-way ANOVA followed by all pairwise multiple comparison procedures (Student–Newman–Keuls method). All data were expressed as mean ± standard error of the mean (SEM). *P* < 0.05 was considered to indicate statistical significance.

## Results

### Effects of different thermal stimulations at LI11 on compound 48/80-induced scratching

**Figure 2:**
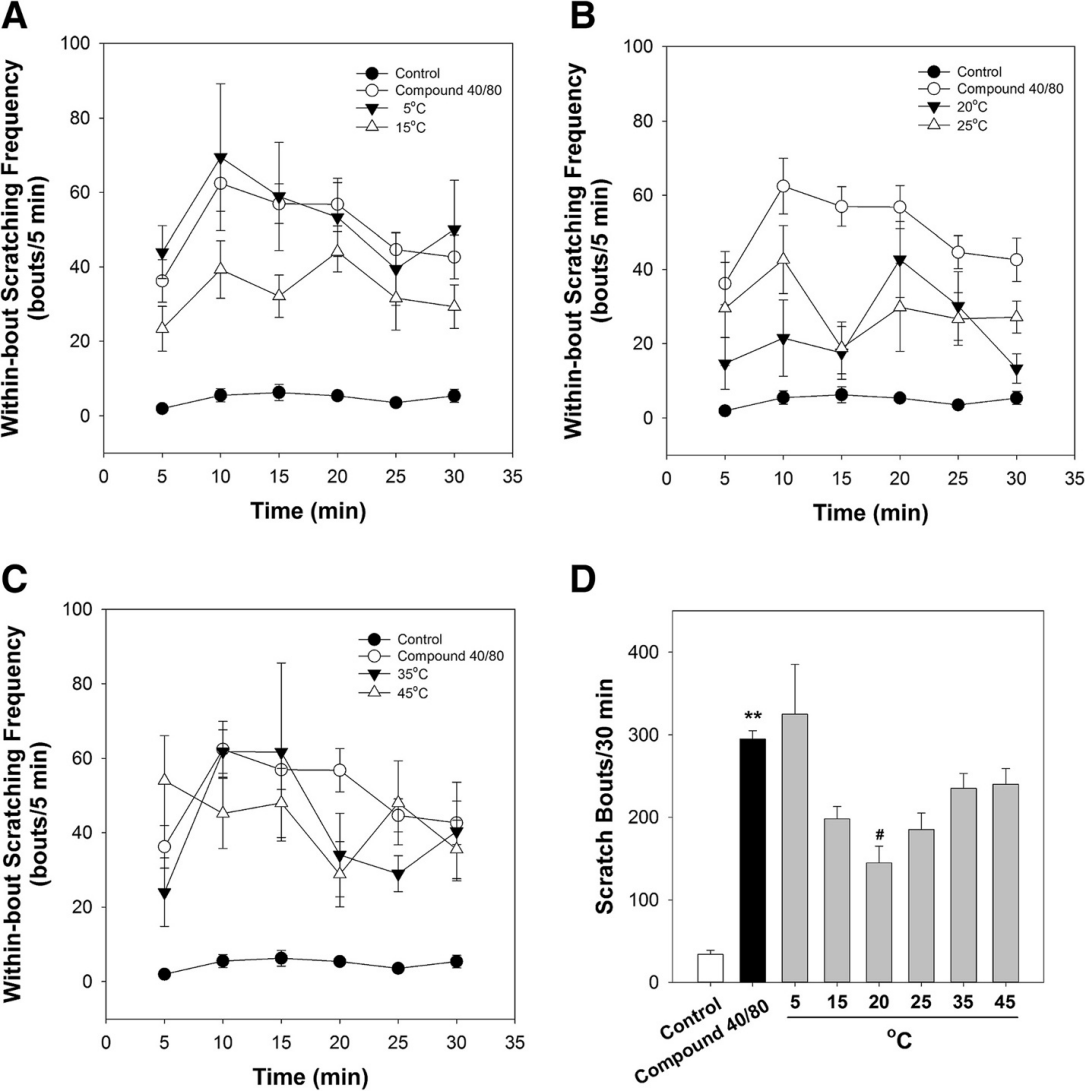
**Effects of different thermal stimulations at LI11 for compound 40/80-evoked scratching.** Both parameters, within-bout scratching frequency **(A)** for 5°C and 15°C, **(B)** 20°C and 25°C, and **(C)** 35°C and 45°C and **(D)** number of scratch bouts, were shown. **P < 0.01 compared to control group. ^#^ *P* < 0.05 compared to compound 40/80 group).

Mice began scratching 3–5 min after injection with compound 48/80. Scratching behaviour peaked 10–20 min after injection with compound 48/80 and continued for 30 min during the observation period. The number of hind leg scratches directed to the back of the neck was counted 5 min intervals for 30 min. Both parameters, within-bout scratching frequency and number of scratch bouts, were shown in Figure 2. Only total counts of scratch bouts were significantly correlated with the concentration of the pruritic stimulus [28].The data show the time course of within-bout scratching frequency for 5°C and 15°C (Figure 2A), 20°C and 25°C (Figure 2B), and 35°C and 45°C (Figure 2C), respectively. The total number of scratches in mice injected with compound 48/80 (290.1 ± 20.9 bouts/30 min) was significantly higher than that in control mice injected with normal saline (28.1 ± 4.4 bouts/30 min). By contrast, the number of scratches in mice treated with 20°C stimulation (139.8 ± 31.9 bouts/30 min), but not 5°C, 15°C, 25°C, 35°C, or 45°C stimulations, at LI11 was significantly decreased (Figure 2D). These results reveal that 20°C is an optimal temperature for cold stimulation at LI11 for attenuating compound 48/80-induced scratching.

### Effect of cold (20°C) stimulation at the sham point did not decrease compound 48/80-induced scratching in mice

**Figure 3:**
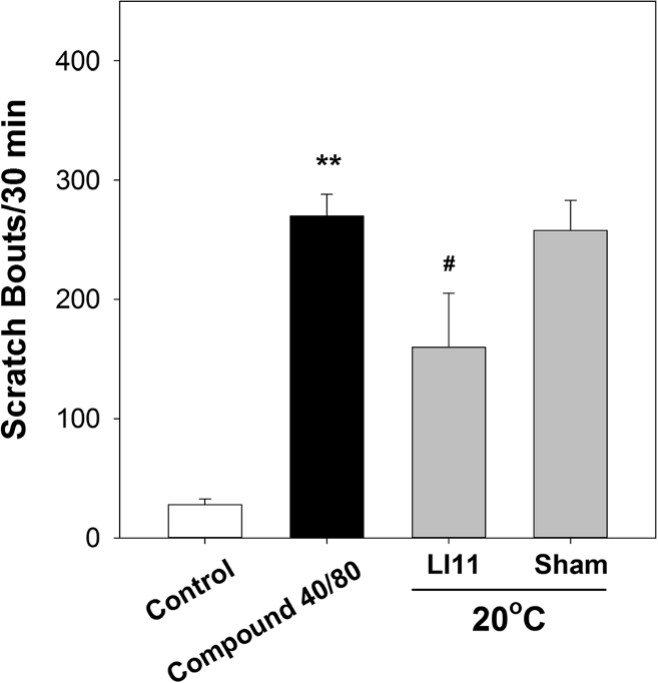
**Evaluating the antiprurtic efficacy of acupoint with a sham (non-acupoint) control.** **P < 0.01 compared to control group. ^#^ *P* < 0.05 compared to compound 40/80 group).

Cold (20°C) stimulation at LI11 decreased compound 48/80-induced scratching in mice. In contrast, the stimulation at the sham points did not decrease scratch counts. The mean number of scratches in mice treated with 20°C stimulation at the sham point (292.3 ± 31.2 bouts/30 min) was similar to that in the compound 48/80 group (Figure 3). This result confirms the specific role for the antipruritic effect of acupoint LI11.

### Cold stimulation at LI11 decreased compound 48/80-induced c-fos expression in the cervical spinal cord

**Figure 4:**
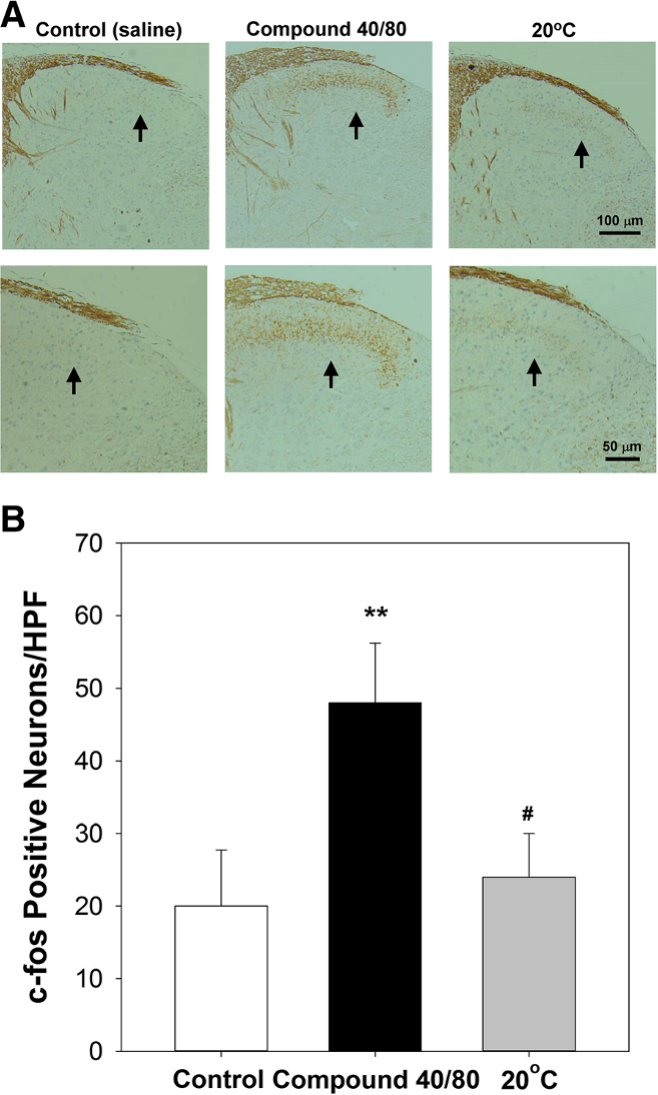
**c-fos expression in the cervical spinal cord. (A)** Representative photomicrographs and **(B)** quantitative results of c-fos expression in the spinal cord. The c-fos-positive neurons were observed under a light microscope at high-power field (HPF) magnification, and the average number of c-fos-positive neurons was counted single-blind using the imaging software. **P < 0.01 compared to control group. ^#^ *P* < 0.05 compared to compound 40/80 group).

The expression of c-fos was increased as compared to control (saline) group in photomicrographs in the lateral side of the superficial lamina of the dorsal horn of the cervical spinal cord following injection with compound 48/80. However, c-fos expression was rarely detected in the superficial layers of the dorsal horn of mice pre-treated with 20°C at LI11 (Figure 4A and 4B). Since itch-related scratching was associated with c-fos expression in the superficial layer of the dorsal horn of the spinal cord. IHC analysis revealed that compound 48/80-induced c-fos expression in the cervical spinal cord was decreased after cold stimulation at LI11.

### Antipruritic effect of cold stimulation at LI11 was decreased by the TRP ion channel blocker

**Figure 5:**
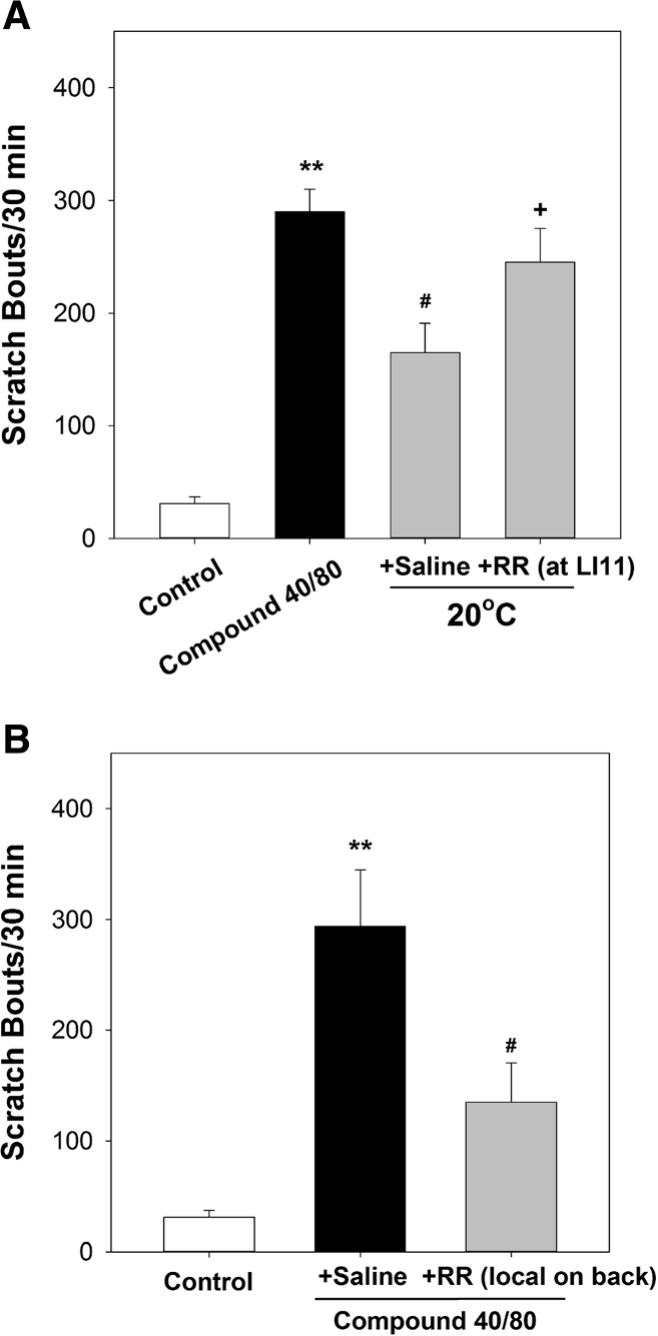
**Evaluating the effect of TRP ion channels blocker (Ruthium red; RR). (A)** Injection with RR at LI11 decreased the antipruritic effect of cold stimulation at LI11. **(B)** Local RR administration with compound 48/80 on mouse back significantly reduced scratching bouts. **P < 0.01 compared to control group. ^#^ *P* < 0.05 compared to compound 40/80 group. ^†^ *P* < 0.05 compared to saline group).

Injection with RR at LI11 decreased the antipruritic effect of cold stimulation at LI11. The mean number of scratches in mice treated with 20°C stimulation at the sham point was similar to that in the compound 48/80 group (Figure 5A).

Compound 48/80 may result in histamine release, and RR (a non-selective TRP antagonist) could reduce histamine-related scratching bouts. We further conducted an experiment with RR and compound 48/80 simultaneous injection on same region on upper back of mice. Interestingly, the result showed that local RR administration with compound 48/80 on mouse back significantly reduced scratching bouts caused by compound 48/80 (Figure 5B). Since RR is a non-selective TRP channel blocker, we suggest that TRP channels may play an important role in the antipruritic effect of cold stimulation at LI11 in mice [37–40].

## Discussion

This study was performed to assess the possible antipruritic effects of different thermal stimulations (from temperatures 5°C to 45°C) at LI11 on compound 48/80-induced scratching behaviour in a murine model. Compared with traditional moxibustion, which uses high thermal stimulation of acupoints, the application of 20°C at LI11 also significantly decreased compound 48/80-induced scratching behaviour in mice. To our knowledge, this is the first report to suggest that cold stimulation at LI11 inhibits pruritogen-induced scratching in an animal model. By contrast, cold stimulation at the sham point did not inhibit scratching behaviour. Pre-treatment with RR at LI11 altered the antipruritic effects of 20°C thermal stimulation at LI11, suggesting that peripheral TRP ion channels may play a role in this effect. Since compound 48/80, a basic polypeptide with potent mast cell degranulating ability, can induce the flare and weal responses after intradermal injected, this pruritogenic agent is conducted in the study of inducing urticaria and the pruritus of atopic dermatitis [41–43].

The mainly thermosensitive afferents express ion channels are the TRP family which respond at distinct temperature thresholds. The different TRP ion channels activation could also altered the perception of itch or pain. For example, TRPA1, TRPM8, TRPV1, and TRPV3 are key channels that transmit the itch sensation [44]; the TRPV1 and TRPV3 play an important role in histamine dependent itch transduction, but the TRPM8 ion channel is hypothesized to inhibit itch signal transmission [27]. Several studies have shown that various TRP channels modulate the itch sensation and therefore may be therapeutically beneficial [44, 45]. Similarities are observed between the neural pathways responsible for itching, pain and temperature, which explains the ability of various temperatures to modulate the desire to scratch. Therefore, TRP channels could serve as important but complex clinical targets for pruritus therapy [46]. In the present study, we suggest the different thermal stimulation acting different TRP ion channels which play important role of the antipruritic effect.

During acupuncture, acupoints are stimulated with needles, which may cause receptors to send neural impulses to the spinal cord or act on the pathways to the brain [47, 48]. This causes the release of neurotransmitters that subsequently modulate functions in the brain and peripheral area [9, 10]. Previous studies have demonstrated that spontaneous itch-related scratching was associated with c-fos expression in the superficial layer of the dorsal horn of the spinal cord in a rat model [49]. Nuclear c-fos can be induced by pruritogens [30]. The localisation of c-fos in the dorsal horn enables individual neurons of animals activated by noxious inputs to be visualised. IHC analysis revealed that compound 48/80-induced c-fos expression in the lateral side of the superficial layer of the dorsal horn at C2–C7 was decreased after 20°C thermal stimulation at LI11.

Pro-inflammatory agents that activate TRP channels in nociceptive neurons can cause neurogenic inflammation and pain. Ceppa et al. reported a major role for TRPV4, which detects osmotic pressure and arachidonic acid metabolites, and TRPA1, which responds to 4-hydroxynonenal and cyclopentenone prostaglandins, in inflammation and pain in mice. Intraductal injection of TRPV4 and TRPA1 agonists increased c-fos expression in spinal neurons, indicative of nociceptor activation, and intraductal TRPA1 agonists also caused inflammation. Deletion of trpv4 or trpa1 gene suppressed c-fos expression and pain behavior. Their results suggest the contributions of TRPV4 and TRPA1 to inflammatory pain [50]. Thus, in this study, c-fos expression on spine is associated with the intense of mice scratch, suggesting it could be altered by the modulation of TRP channels.

Limitations of the current study must be acknowledged. The study was performed using only one pruritogen, which induced murine scratching by mainly histamine-dependent pathway, additional pruritogens that activate different scratching signal impulse pathways should be investigated. Moreover, we showed that injection with RR at LI11 decreased the antipruritic effect of cold stimulation at LI11. It should be further clarified what happened to c-fos in the cervical spinal cord after injection with RR at LI11 in future works (schematic representation in Figure 1A). A clinical trial performed on patients with pruritic dermatitis treated with cold stimulation at LI11 may also confirm that cryo-acupuncture is effective in humans.

## Conclusions

Our findings revealed that 20°C cold stimulation at LI11 attenuated compound 48/80-induced scratching in mice, suggesting that the efficacy of acupoint stimulation can be achieved not only by traditional needles or moxibustion but also by cold stimulation. Therefore, cold stimulation at LI11 is promising as a potentially useful novel non-invasive alternative antipruritic treatment in humans.

## Abbreviations

LI11: Quchi acupoint
RR: Ruthium red
s.c.: Subcutaneous
TRP: Transient receptor potential
IHC: Immunohistochemistry
i.p.: Intraperitoneal
SEM: Standard error of the mean.
